# Protein-Coated Nanoparticles Are Internalized by the Epithelial Cells of the Female Reproductive Tract and Induce Systemic and Mucosal Immune Responses

**DOI:** 10.1371/journal.pone.0114601

**Published:** 2014-12-09

**Authors:** Savannah E. Howe, Vjollca H. Konjufca

**Affiliations:** Department of Microbiology, Southern Illinois University, Carbondale, Illinois, United States of America; Midwestern University, United States of America

## Abstract

The female reproductive tract (FRT) includes the oviducts (fallopian tubes), uterus, cervix and vagina. A layer of columnar epithelium separates the endocervix and uterus from the outside environment, while the vagina is lined with stratified squamous epithelium. The mucosa of the FRT is exposed to antigens originating from microflora, and occasionally from infectious microorganisms. Whether epithelial cells (ECs) of the FRT take up (sample) the lumen antigens is not known. To address this question, we examined the uptake of 20–40 nm nanoparticles (NPs) applied vaginally to mice which were not treated with hormones, epithelial disruptors, or adjuvants. We found that 20 and 40 nm NPs are quickly internalized by ECs of the upper FRT and within one hour could be observed in the lymphatic ducts that drain the FRT, as well as in the ileac lymph nodes (ILNs) and the mesenteric lymph nodes (MLNs). Chicken ovalbumin (Ova) conjugated to 20 nm NPs (NP-Ova) when administered vaginally reaches the internal milieu in an immunologically relevant form; thus vaginal immunization of mice with NP-Ova induces systemic IgG to Ova antigen. Most importantly, vaginal immunization primes the intestinal mucosa for secretion of sIgA. Sub-cutaneous (s.c) boosting immunization with Ova in complete Freund's adjuvant (CFA) further elevates the systemic (IgG1 and IgG2c) as well as mucosal (IgG1 and sIgA) antibody titers. These findings suggest that the modes of antigen uptake at mucosal surfaces and pathways of antigen transport are more complex than previously appreciated.

## Introduction

The mucosa of the FRT is a major site of entry and transmission of sexually transmitted pathogens such as *Chlamydia*, *Gonorrhea*, human immunodeficiency virus (HIV), human papillomavirus (HPV), etc.. In the U.S. alone, about 20 million new sexually-transmitted infections (STIs) occur annually, with the highest rates amongst young people in their reproductive prime (15–30 years of age) [1]. In spite of research efforts, the development of mucosal vaccines against STIs has generally been unsuccessful with the lone exception being parenteral vaccines against human papillomavirus (HPV), which induce high systemic antibody titers and protect against HPV challenge [2]. IgG and IgA antibodies secreted at mucosal surfaces protect against toxins, as well as bacteria and viruses [3], [4], [5]. Both systemic and local IgG antibodies are also important for protection against HIV, as demonstrated in rhesus macaques, which were protected against a vaginal challenge with SHIV when HIV-specific IgG antibodies were administered either systemically or intra-vaginally [6], [7], [8]. IgG antibodies were shown to bind to and neutralize the virus, thus preventing its entry into the host via the genital tract. Therefore, the efficacy of a vaccine that targets STIs will in great part depend on the vaccine's ability to induce production of antibodies at mucosal surfaces, in addition to systemic antibodies. While humoral immunity alone protects against some pathogens, induction of both cell-mediated and humoral immunity locally via intra-vaginal immunization is necessary for protection against pathogens such as *C. trachomatis*, *N. gonorrhea*, herpes simplex virus (HSV), HIV, etc. [9], [10], [11], [12]. Unlike the mucosa of the intestinal tract, mucosa of the FRT does not contain organized lymphoid tissues, such as Peyer's Patches (PP) or microfold cells (M cells) that that are important for the uptake of lumen antigens and for induction of immune responses. In the intestinal mucosa, soluble and particulate antigens can enter the internal milieu via M cells [13], [14], [15], goblet cell associated pathways (GAPs) [16], [17], lamina propria dendritic cells [18], [19], [20], and ECs [17]. Whether ECs of the FRT play a role in the uptake (sampling) of mucosal antigens is not known. ECs of the FRT are covered with a layer of mucus that prevents their direct contact with the lumen antigens originating from microflora or from infectious microorganisms. It is generally thought that the mucosa of the FRT is a poor site for induction of immune responses and very little is known about the modes of vaccination that would maximize both local and systemic immune responses [21]. However, it is known that immunization via this route is necessary for the induction of a local immune response. Intra-vaginal immunizations with non-infective vaccine formulations such as soluble antigens or inactivated poliovirus induce weak local and rarely systemic humoral immune responses, possibly because they do not reach the immune cells in the sub-mucosa of the FRT efficiently [22]. Infections with *N. gonorrhoeae*, *C. trachomatis*, group B streptococci, HSV type 2, or HPV, can induce systemic antibodies, but induce weak local antibody responses [22], [23]. In a Phase I clinical trial, vaginal immunization with HIV-1 gp140 antigen without an adjuvant failed to induce sustained systemic or local IgG, IgA, or T cell responses in women [23]. Studies conducted in animals and in humans show that intra-vaginal immunizations with antigens, co-administered with toxin-based adjuvants, (such as cholera toxin B) can induce immune responses in the FRT [24], [25]. However, safety concerns associated with toxin-based adjuvants necessitate development of vaccine formulations and prime-boost vaccination protocols that can induce both local and systemic immunity without the use of adjuvants. To enhance antigen entry in the FRT mucosa without the use of adjuvants, in many studies pre-treatment of animals with progesterone, followed by mechanical or chemical disruption of the FRT epithelium is commonly practiced. These approaches were used for vaginal delivery of PLGA NPs [28], virus-like particles (VLP), HPV [12], and quantum dots (<10 nm) [29]. However, progesterone treatment was shown to affect both susceptibility to infections and immune responses in vaccinated animals [26], [27]. In addition, the use of epithelial disruptors would interfere with examining whether ECs of the FRT sample lumen antigens. We have shown that intestinal ECs can internalize NPs (20-40 nm in diameter), which are then transported to the mesenteric lymph nodes (MLNs) that drain the intestine [17]. Here, using 20 and 40 nm NPs as a model particulate antigen, we show that NPs administered vaginally are internalized by the vaginal ECs, as well as ECs of the upper FRT. In addition, NPs are transported to the ILNs, MLNs, and the serosa of the large intestine. We show that intra-vaginal immunization of mice with Ova-conjugated 20 nm NPs, in combination with a s.c. boost, induces systemic as well as mucosal antibodies. In these studies animals were given no progesterone, the epithelium was not disrupted by any means, and no adjuvants were used, indicating that the FRT mucosa, much like the mucosa of the small intestine, has mechanisms by which antigens from the FRT lumen are internalized and transported to the internal milieu. Our findings have implications for understanding the biology of the FRT, as well as for development of mucosal vaccines to target STIs.

## Materials and Methods

### Ethics statement

This study was carried out in strict accordance with the recommendations in the Guide for the Care and Use of Laboratory Animals of the National Institutes of Health. The protocol was approved by the Southern Illinois University Institutional Animal Care and Use Committee (Protocol Number: 13-057). Animals were housed in centralized AAALAC-accredited research animal facilities, staffed with trained husbandry, technical, and veterinary personnel.

### Animals, model antigens, reagents, and antibodies

For these studies six to eight week-old female C57BL/6 mice (Jackson Laboratories) were used. Chicken Ova (Sigma) was used as a model protein antigen. Carboxylate-modified fluorescent polystyrene nanoparticles (20 nm and 40 nm, Invitrogen), were used as a model particulate antigen, as well as Ova antigen carriers for immunizations. For immunization experiments 20 nm NPs were conjugated to Ova and every batch of conjugated NPs was analyzed by dot-blot as described previously [17]. Biotinylated rabbit anti-Ova antibodies (Thermo Scientific) in combination with streptavidin-FITC (eBioscience) were used to detect Ova antigen and NP-Ova. To highlight the tissue architecture in tissue cryosections, actin-binding Phalloidin-Alexa350 (Invitrogen) was used. Tissue staining with biotinylated Lyve-1 (eBioscience) and E-cadherin (BD Biosciences) antibodies, followed by FITC-conjugated streptavidin (eBioscience) was done in order to visualize the lymphatics and the FRT epithelium. Some tissue sections were stained with antibodies specific for CD11c+ DCs (eBioscience). All antibodies were used at a 1∶100 dilution in blocking buffer.

### Administration of NP-Ova to the mice

Mice were instilled vaginally with 10–20 µl of 20 nm NP-Ova (20% diluted in PBS from an original 2% concentrated stock solution) or PBS (control) on days 0, 1, 3, 5, and 7. The total amount of Ova administered vaginally via NPs was 100–150 µg. For this, mice were anesthetized lightly with isofluorane delivered in a stream of oxygen. NP-Ova or PBS solution were administered into the vaginal canal using a 20 µl pipette. After NP-Ova administration, excess NP-Ova solution was aspirated and the vaginal opening was thoroughly wiped with cotton swabs soaked in 70% ethanol in order to preclude the per-oral sampling of NPs via vaginal grooming. In another experiment, anesthetized mice were administered 5–10 µl of NP-Ova on days 0, 1, and 2. After NP administration and wiping of the vaginal opening with ethanol-soaked cotton swabs, mice were fitted with Elizabethan neck collars and placed in separate cages for up to one week after immunization in order to preclude per-oral sampling of NP-Ova via grooming. On day 28 after the first vaginal NP-Ova or PBS administration, mice were s.c. injected with 300 µg Ova in CFA (Sigma).

### Analysis of NP uptake in the FRT and their subsequent transport to other tissues by immunofluorescence microscopy (IFM) and confocal microscopy

The internalization of NPs in the FRT and their transport to the draining ILNs, MLNs, and the large intestine was examined by immunofluorescence microscopy (IFM) and confocal microscopy. For these experiments mice were anesthetized with isoflurane delivered in a stream of oxygen, then instilled intra-vaginally with 10–20 µl of 20 or 40 nm red fluorescent NPs diluted in PBS (10–20%). For examining NP uptake in the FRT and transport to the lymph nodes and the large intestine, mice were kept under light anesthesia for the duration of the experiments. At different times post NP instillation, mice were euthanized and the ILNs, MLNs, FRT, and the large intestine were excised and snap-frozen in Tissue-Tek® O.C.T. freezing compound on dry ice. Tissue cryosections (5–7 µm thick) were fixed in 4% paraformaldehyde (PFA), washed with PBS then incubated with blocking buffer (Thermo Scientific) for 10–15 minutes. Tissue sections were then stained with fluorescently-tagged antibodies and imaged with a Leica DM4000B fluorescent microscope. Acquired images were analyzed using Volocity software. Excised MLNs and ILNs from two experiments (conducted as described above) were imaged with a confocal microscope as described previously [17].

### Collection of vaginal washings, fecal pellets, and blood samples

Before vaginal instillation of NP-Ova or PBS, and every week thereafter, vaginal washings were collected in 100 µl of PBS. In one study, vaginal washes were collected in 20 µl of PBS over three days. Blood was collected via the tail vein using a 30 g needle and serum was separated. Fecal pellets were collected from each mouse and diluted in PBS containing 0.02% sodium azide (Sigma) to a final concentration of 100 mg dry matter/ml of PBS. Diluted fecal pellets were homogenized and then centrifuged at 10,000×g for 10 minutes. All collected samples were stored at −20°C until assayed.

### Determination of Ova-specific antibody titers in vaginal washings, fecal pellets, and serum samples using ELISA assay

For ELISA assays starting sample dilutions were from 1∶10 (for fecal extracts and vaginal washings) to 1∶50 and 1∶100 for serum samples prior to boosting. After boosting immunization starting dilutions for serum samples were up to 1∶1000. Flat-bottomed 96-well plates were coated with 100 µl of 50 µg/ml Ova (Sigma) solution in coating buffer (0.02 M Na_2_CO_3_/0.07 M NaHCO_3_ in H_2_O, pH 9.6) and allowed to incubate overnight at 4°C. After the removal of unbound antigen, plates were blocked for 1 h at 37°C with 200 µl of blocking buffer (0.2% porcine gelatin (Sigma) in PBS). Plates were then washed with 200 µl/well of PBS with 0.05% Tween-20 (Sigma) and 0.02% sodium azide (Sigma) three times using an automated plate washer (BioTek, ELx50). After washing, 200 µl of sample (serum, fecal extract or vaginal washings, diluted in blocking buffer) were added the first column of wells and then diluted into successive wells of blocking buffer and allowed to incubate overnight at 4°C. After overnight incubations, plates were washed three times and 100 µl of alkaline phosphatase-conjugated goat anti-mouse IgG1, IgG2c, or IgA (Southern Biotech), diluted 1∶2000 in blocking buffer, were added to each well and allowed to incubate for 2 h at room temperature. Plates were then washed three times, and alkaline phosphatase (AP) activity was assayed by adding 100 µl of 1 mg/ml AP substrate (Sigma) and incubating for 20 minutes at room temperature, protected from light. The reaction was stopped with 25 µl of 3 M NaOH, and the absorbance was read at 405 nm using a plate reader (BioTek, Epoch).

### Detection of Ova-specific antibodies by western blot analysis

For this 1 µl of Ova (10 mg/ml) or PBS (control) was spotted onto a 0.2 µm nylon membrane and allowed to dry. The membrane was washed with Tris-buffered solution containing 0.1% Tween 20 (Sigma) (TTBS), blocked with TTBS containing 5% skim milk (Difco) for 1 h, then incubated with either serum, fecal extracts, or vaginal washes diluted in PBS (1∶100 for sera and 1∶50 for mucosal washes) overnight at 4°C. After 3 washes, the membranes were incubated with AP-conjugated goat anti-mouse IgG1or IgA for 1 hour, washed with TTBS, and the immunoreactive dots were detected by the addition of nitroblue tetrazolium (NBT)-5-bromo-4-chloro-3-indolylphosphate (BCIP) (Sigma). The reaction was stopped after 2 to 5 min by washing the membranes with deionized water.

### Statistical analysis

Each experiment was repeated at least three times. Antibody titers were expressed as log_10_ of the highest sample dilution that yielded an OD_405_ value twice that of the negative control. Data were analyzed using ANOVA procedures of SAS software. Group means were separated using Student's t-test and were considered significantly different at P<0.05. Data are expressed as the mean ± standard deviation of the mean.

## Results

### NPs are internalized by ECs of the upper and lower FRT and reach the draining ILNs, MLNs, and the large intestine

**Figure 1:**
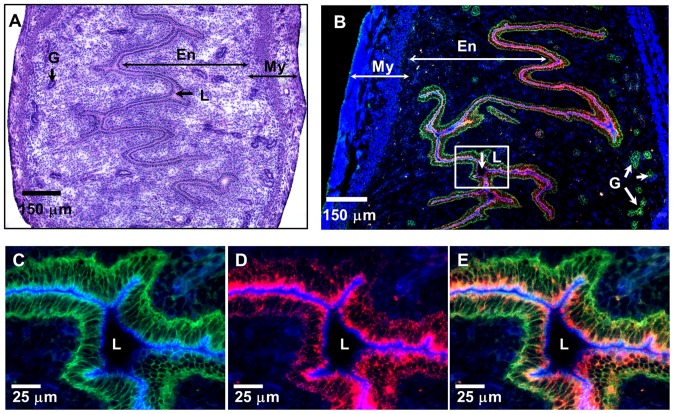
Vaginally applied NPs reach the lumen of the uterus and are internalized by uterine ECs. (A) H&E stained tissue section of a mouse uterus; (B) An IFM image of the uterine tissue section 3 h after intra-vaginal administration of 20 nm fluorescent NPs (red). (C, D) Two-color and (E) three color higher magnification IFM image of the uterine epithelium shown in the boxed inset from panel B. (B–E) Tissue sections were stained with actin-binding phalloidin-Alexa350 (blue) and anti-E-cadherin antibodies (green), while fluorescent NPs are shown in red. En-endometrium, My-myometrium, L-lumen, G- mucus glands of the uterus.

**Figure 2:**
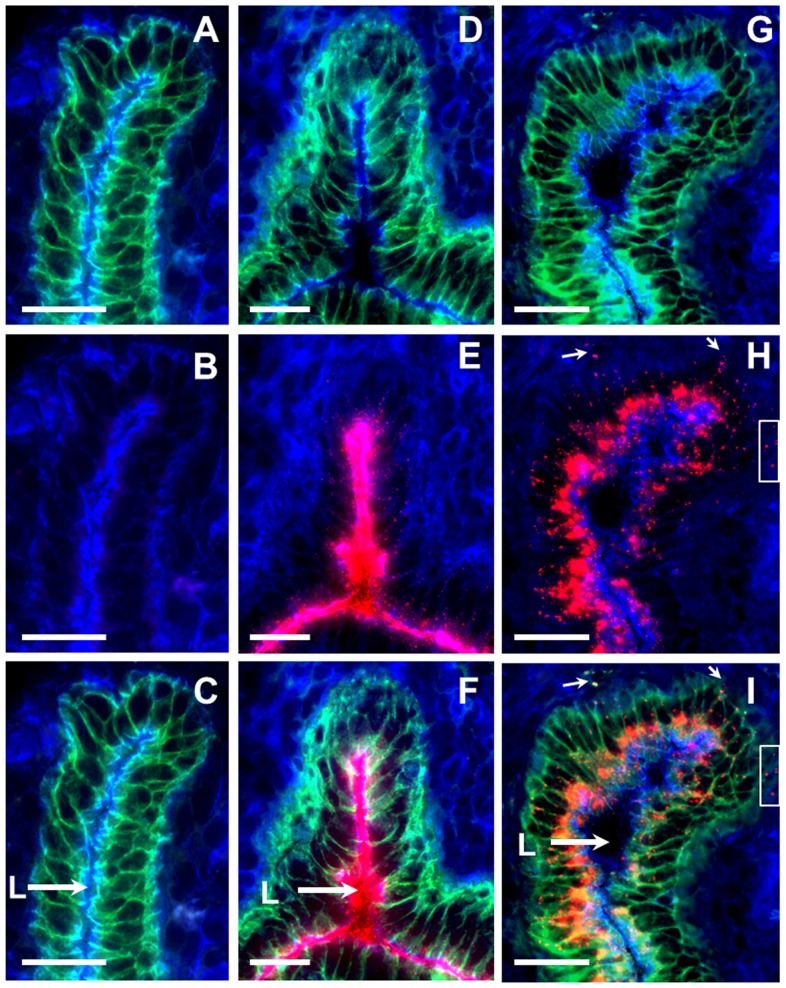
Internalization of 20 nm NPs by uterine ECs at 1 h and 6 h after intra-vaginal administration. (A-C) Two- and three-color IFM images of uterine epithelium from a control mouse. (D–F) Two- and three-color images of the uterine epithelium at 1 h and (G–I) 6 h after intra-vaginal administration of 20 nm NPs. (H, I) NP clumps on the basolateral side of the epithelium (box, arrows). Tissue sections were stained with actin-binding phalloidin-Alexa350 (blue) and anti-E-cadherin antibodies (green), while fluorescent NPs are shown in red. Scale bar = 25 µm, L-lumen.

In initial experiments we examined whether ECs of the FRT can internalize (sample) NPs administered in the lumen without treatment with progesterone, EC disruptors, or mucosal adjuvants. We found that NPs (20 and 40 nm) instilled vaginally can reach the upper FRT (uterus) and are subsequently internalized by E-cadherin expressing ECs (Fig. 1 A–E). We then examined the dynamics of NP internalization by uterine ECs. As expected, no red fluorescence was observed in the lumen or ECs of the control mice (Fig. 2 A–C). Within 1 h of administration, NPs were observed co-localizing with E-cadherin+ uterine ECs (Fig. 2 D–F), however a significant amount of NPs remained within the lumen (L) of the uterus (Fig. 2 E, F, arrow). At later time points (6–12 h) after vaginal administration, most of the NPs were observed within the ECs, as well as in the sub-epithelial compartment (Fig. 2 H, I, box, small arrows), while a small fraction of NPs were seen in the lumen (L) (Fig. 2 G–I, large arrow), indicating a time-dependent NP internalization. In addition, NPs were also observed within the lamina propria and uterine serosa (S1 A–C Figure).

**Figure 3:**
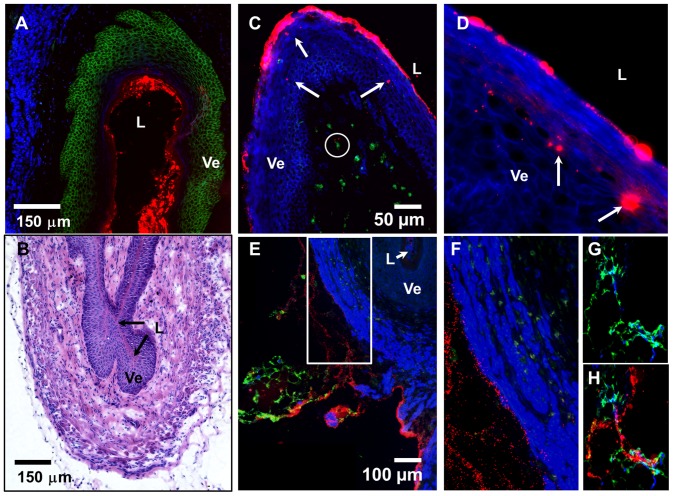
NPs instilled vaginally are internalized by vaginal ECs and drain from the FRT via the lymphatics. (A) A three color IFM image of a vaginal tissue section. (B) An image of the H&E stained vaginal tissue section. (C) A three-color IFM image of the vaginal tissue section depicting red NPs within the vaginal epithelium (Ve) (arrows), and in the sub-epithelial compartment (circled) near CD11c+ DCs (green). (D) A two-color IFM high magnification (630x) image of the vaginal epithelium (blue) harboring internalized NPs (red). (E) A three color IFM image of a vaginal tissue section showing the NPs (red) draining from the vaginal tissue via the lymphatics of the FRT (green). (F) A three-color high magnification image of the boxed inset from panel E showing NPs (red) draining from the FRT. (G, H) Two- and three-color IFM images of lymph ducts draining from the FRT and carrying NPs (red). (A, C–H) Vaginal tissue sections were stained with actin-binding phalloidin-Alexa350 (blue), in combination with (A) E-cadherin (green), (C) CD11c (green), and (E, H) Lyve-1 (green) antibodies, while fluorescent NPs are shown in red. (A–E) Ve-vaginal epithelium, L-vaginal lumen.

NPs were also internalized by the vaginal ECs (Fig. 3 A, B), and could be observed as bright clumps within the vaginal epithelium (Ve) (Fig. 3 C, D, arrows). In addition, NPs reached the sub-epithelial compartments and small NP aggregates were observed in proximity of CD11c+ DCs (Fig. 3 C, circled). Within 1 h of administration, NPs could be seen in the serosa of the FRT tissue sections and co-localizing with Lyve-1+ lymphatic ducts that drain the FRT (Fig. 3 E–H). In general, the degree of internalization of NPs by the vaginal ECs was more variable.

**Figure 4:**
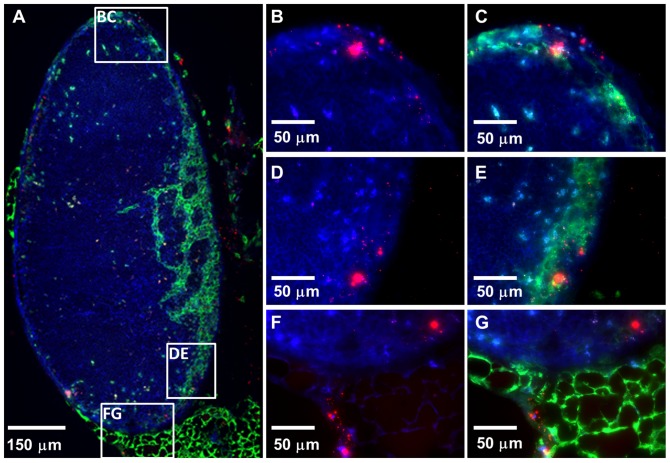
NPs distributed within an ILN 5 h after intra-vaginal administration. (A) A three-color stitched IFM image showing an ILN harboring NPs (red). Two- and three-color higher magnification (630x) IFM images of the top (B, C), middle (D, E) and bottom (F, G) box insets from panel A showing 20 nm NPs (red) within the ILN. Tissue sections were stained with actin-binding phalloidin-Alexa350 (blue) and Lyve-1 antibodies (green), while fluorescent NPs are shown in red.

**Figure 5:**
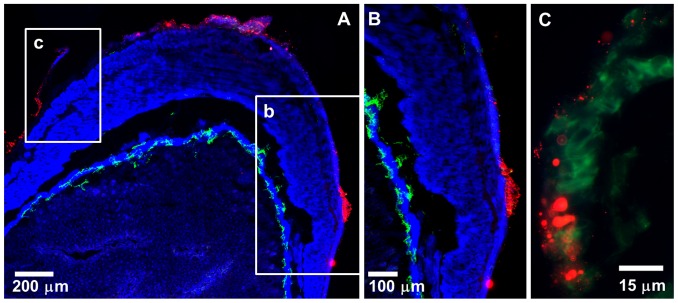
NPs administered intra-vaginally transport to the serosa of the large intestine. 10–20 µl of NPs (diluted to 20% in PBS) were administered vaginally to an anesthetized mouse. Mouse was maintained under light anesthesia for the duration of the experiment (3 h) at which time the mouse was euthanized and the large intestine was excised and snap-frozen for IFM analysis. (A) A low magnification stitched IFM image of a section of the large intestine showing 20 nm NPs (red) in the intestinal serosa. (B, C) High magnification (630x) IFM images of the boxed insets (B, C) from panel A. A tissue section of the large intestine was stained with actin-binding phalloidin-Alexa350 (blue) and Lyve-1 antibodies (green), while NPs are shown in red.

NPs also transported to the ILNs that drain the FRT 1 h after administration and clumps of accumulated NPs were apparent within ILNs by 3–5 h after administration (Fig. 4 A–G). NPs were found co-localizing with the Lyve-1^+^ regions of the ILNs, indicating their association with the lymphatic flow (Fig. 4 A, C, E, G). Interestingly, NPs also reached the MLNs that drain the intestinal tract within 1 h of their vaginal instillation and were observed within the adipose tissue surrounding the MLNs (S2A Figure) and within the MLNs (S2B Figure). At later times after administration, clusters of NPs were observed within the MLN tissue sections by IFM (S2C Figure). We then examined the tissue sections of the large intestine collected from mice to which NPs were administered intra-vaginally. Surprisingly, NPs were observed within the serosa of the large intestine (Fig. 5 A–C), indicating that there is some mode of transport that allows vaginally-administered NPs to reach the large intestine.

### Vaginal immunization with Ova-conjugated NPs induces systemic IgG1 and IgG2c antibody titers, which can be boosted by s.c. immunization

**Figure 6:**
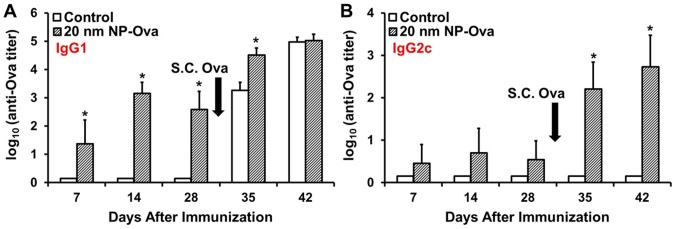
Antibody titers in sera of mice following priming vaginal immunization with 20 nm NP-Ova and s.c. boosting immunization with Ova in CFA. Mice were immunized vaginally with 20 nm NP-Ova or PBS (control), then s.c. boosted with 300 µg Ova with CFA at day 28 after priming immunization (arrows). (A) Ova-specific IgG1 and (B) IgG2c antibody titers are expressed as log_10_ titer values, with the titer being the highest dilution that yielded an OD_405_ absorbance value two times that of the negative control. Values represent the mean ± SD of samples collected from 14 mice per group (3 separate experiments). Group means were separated using Student's t-test and declared significantly different at a P<0.05. Asterisks indicate significant differences between treatment means.

We next examined whether vaginal immunization with NP-Ova would elicit humoral immune responses in immunized mice. For this, anesthetized mice were vaginally immunized with 20 nm NP-Ova (or PBS), then at day 28 after initial immunization mice were s.c. immunized with Ova in CFA. A few points are important to emphasize: 1) Mice were instilled with 10–20 µl of NP-Ova; 2) Any excess NPs “leaking” from the vaginal tract were aspirated and the opening of the vaginal tract was carefully wiped with cotton swabs; 3) In every experiment, about 1/3rd of the mice did not develop titers measurable by ELISA, which we termed “non-responders”. In the “responder” mice, vaginal immunization with NP-Ova induced Ova-specific IgG1 and IgG2c antibodies in sera of immunized mice at day 7, 14, and 28 post-immunization (Fig. 6 A, B). At day 7, 7 of 14 mice were negative for Ova-specific serum IgG1 at 1∶50 dilution, however they were positive for IgG1 when tested by dot blot (not shown), indicating that the Ova-specific titers were very low. By week 2 and 3, all 14 mice developed significant serum IgG1 titers. This s.c. immunization with Ova+CFA at day 28 significantly boosted the IgG1 and IgG2c serum antibody titers (Fig. 6 A, B). No IgG1 or IgG2c was detected in sera of mice administered PBS vaginally (control mice) at days 7, 14, and 28 (Fig. 6 A, B). After s.c. immunization with Ova+CFA, control mice exhibited elevated serum IgG1 titers, which were comparable to the titers of the vaginally-primed mice by day 42 (Fig. 6 A). However, control mice immunized only s.c. had no serum IgG2c titers, indicating that vaginal immunization with NP-Ova was needed for induction of IgG1 and IgG2c antibody titers (Th1/Th2 immune response).

### Vaginal immunization with NP-Ova induces secretion of sIgA in the intestinal mucosa

**Figure 7:**
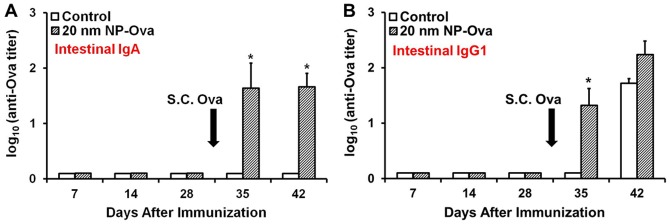
Antibody titers in fecal extracts of mice vaginally primed with 20 nm NP-Ova (or PBS, control) and s.c. boosted with 300 µg Ova with CFA at day 28 after priming immunization (arrows). Fecal pellets were collected each week from individual mice and fecal extracts were assayed by ELISA. (A) Ova-specific sIgA titers in fecal extracts. (B) Ova-specific IgG1 titers in fecal extracts. Antibody titers are expressed as log_10_ titer values, with the titer being the highest dilution that yielded an OD_405_ absorbance value two times that of the negative control. Values represent the mean ± SD of samples collected from 14 mice per group (3 separate experiments). Group means were separated using Student's t-test and declared significantly different at a P<0.05. Asterisks indicate significant differences between treatment means.

**Figure 8:**
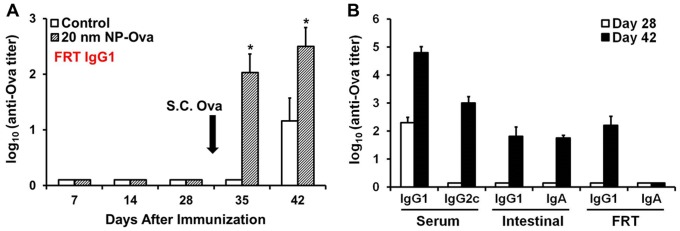
Antibody titers of mice vaginally-primed with 20 nm NP-Ova or PBS (control) and s.c. boosted with 300 µg Ova with CFA at day 28 after priming immunization (arrow). (A) IgG1 antibody titers in vaginal washings. Antibody titers are expressed as log_10_ titer values, with the titer being the highest dilution that yielded an OD_405_ absorbance value two times that of the negative control. Values represent the mean ± SD of samples collected from 14 mice per group (3 separate experiments). Group means were separated using Student's t-test and declared significantly different at a P<0.05. Asterisks indicate significantly differences between treatment means. (B) Serum, intestinal, and FRT antibody titers of mice that were fitted with Elizabethan neck collars. Values represent the mean ± SD of samples collected from 6 mice per group.

In addition to PP, MLNs are also important sites where antibody diversification via V(D)J somatic hypermutation and class switch recombination occurs [30]. Based on our observation that vaginally-administered NPs were transported to the MLNs and the large intestine, we hypothesized that this would lead to imprinting of gut-specific homing of B cells and IgA class switching. At days 7, 14, and 28 post-vaginal immunization no significant sIgA was detected in fecal extracts of control or NP-Ova immunized mice (Figs. 7 A, 8 B), however s.c. immunization with Ova+CFA boosted the sIgA titers (Fig. 7 A, 8 B). This finding indicates that vaginal immunization did prime the intestinal mucosa for secretion of sIgA and IgG1, which became apparent after s.c. boosting immunization (Fig. 7, 8 B). The fact that we could not detect sIgA and IgG1 in fecal extracts after priming immunization is not surprising, since it is very likely that the amount of sIgA in fecal pellets was too low.

### Vaginal immunization with NP-Ova induces secretion of IgG1, but not sIgA in the vaginal mucosa

We did not find a significant amount of IgG1 in the vaginal washings at days 7, 14, and 28 (Fig. 8) in spite of presence of serum IgG1 (Fig. 6 A). S.c. immunization led to a drastic increase in the amount of IgG1 in the vaginal washings of both vaginally-primed and control mice (Fig. 8 A). However, vaginally-primed mice had significantly higher IgG1 compared to control mice at day 35 and 42 (Fig. 8 A), although the serum IgG1 titers were comparable between these groups after s.c. boost (Fig. 6 A). Others have shown that IgG is produced by local B cells of the vaginal mucosa [31], in addition to being secreted from the serum into the FRT lumen. We did not detect sIgA in vaginal washings at any time point using ELISA assay (Fig. 8 B).

To preclude the possibility that observed immune responses in “responders” were not due to ingestion of NP-Ova via grooming we conducted an additional experiment in which mice were intra-vaginally immunized with 5–10 µl of NP-Ova at days 0, 1, and 2. After each NP-Ova application, the vaginal opening was cleaned as described above, then mice were fitted with neck cones (Elizabethan collars) and kept in individual cages for up to one week after vaginal NP administration in order to prevent grooming. Similar to previous results, immunized mice exhibited serum IgG1 titers, but no serum IgG2c or mucosal IgG1 and IgA antibodies at day 28 after intra-vaginal immunization (Fig. 8 B). S.c. injection with Ova+CFA boosted serum IgG1 and IgG2c, intestinal IgG1and sIgA, and FRT IgG1 antibodies (Fig. 8 B). However, we failed to detect sIgA in the FRT washings by ELISA, although washings were collected in 20 µl of PBS (Fig. 8 B). An important point that needs to be addressed is that in only half of the animals (3/6) used in this study, serum IgG1 antibody titers were measurable by ELISA (at a 1∶50 dilution) at day 7 and 14 after vaginal priming. However, Ova-specific IgG1 was detected in sera of all 6 mice by day 7 after vaginal immunization by dot blot analysis (S3 Figure), indicating that there is variability in the amount of NP internalization and thus the ensuing immune responses among animals. By day 28 all 6 serum IgG1titers could be measured in all 6 mice. Intestinal sIgA was measurable by ELISA in animals that had high IgG1/IgG2c titers at day 42 (3/6). However, sIgA was detected in fecal extracts of all 6 animals by western blot analysis (S3 Figure). sIgA in vaginal washings was detected by western blot analysis at 1 week after priming (not shown), however unlike the intestinal sIgA, the vaginal sIgA titers were not boosted after s.c. injection with Ova+CFA.

## Discussion

Immunization via the FRT mucosa is the preferable route for induction of local immune responses which are essential for prevention of STIs and for limiting disease severity [32], [33], [34]. However, vaginal immunization is challenging for a number of reasons. The permeability and sloughing of the FRT epithelium is under hormonal control, thus the uptake and immunogenicity of vaginally-administered vaccine formulations can vary drastically depending on the estrus stage of the immunized animals. Not much is known about the mechanisms of antigen uptake in the FRT and subsequent transport to the deeper lymphoid tissues. Based on the finding that ECs of the small intestine can internalize 20 and 40 nm NPs [17], we hypothesized that ECs of the FRT could also internalize NPs and that NPs could be an effective vehicle for antigen transport from the FRT lumen to the deeper lymphoid tissues. In this work we show that 1) ECs of the FRT internalize 20 and 40 nm NPs; 2) internalized NPs are transported to ILNs that drain the FRT, large intestine, and the MLNs that drain the intestinal tract; 3) model protein antigen Ova conjugated to NPs reaches the lymphoid tissues in an immunogenic form and induces systemic antibody responses; and 4) vaginal immunization with NP-Ova primes the intestinal mucosa for secretion of sIgA. In addition, we demonstrate that s.c. immunization of vaginally-primed mice further boosts systemic as well as mucosal antibody responses. We took a number of measures in order to restrain the mice from grooming the vaginal area and to prevent oral inoculation with NP-Ova. In addition, since a very small amount of NPs was used for immunizations, it is unlikely that a sufficient amount of NP-Ova was sampled per-orally that would explain the induction of sIgA in intestinal secretions. Moreover, even if some NP-Ova was sampled per-orally in initial experiments in which restraint collars were not used, most likely the small amount of Ova present in these NPs would have been degraded by the gastric acidity and intestinal digestive enzymes before reaching the intestinal lymphoid tissues. In addition, the humoral immune responses observed in the study in which neck collars were used to preclude NP-Ova sampling via grooming confirmed the initial immunization results, including a high degree of variability in the magnitude of the humoral immune responses, which is not observed when mice are immunized s.c..

We argue that this variability stems from differences among mice in the amount of internalized NP-Ova, although quantifying the actual dose (amount) of Ova antigen that was internalized by the FRT mucosa is not possible. In addition, quantification of antibodies in mucosal secretions is challenging in part because antibodies are diluted in the washing buffer or in the fecal material. In spite of this, we consistently detected sIgA and IgG1in fecal extracts and vaginal washes, respectively. It is well established that IgG represents a dominant antibody isotype in the FRT secretions [35], [36] and it is believed that serum IgG passively enters the FRT by transudation [21], but is also produced locally by mucosal B lymphocytes [31]. We could not detect IgG1 by ELISA in vaginal washes prior to boosting immunization, although serum IgG1 titers were quite high. However, after s.c. boosting immunization, IgG1 titers in vaginal washings of NP-Ova immunized mice were higher compared to controls, indicating a local production of IgG1. Interestingly, in all experiments we failed to observe measurable concentrations of sIgA in the FRT secretions, although Ova-specific sIgA could be detected by western blot analysis. Unlike intestinal mucosa, FRT mucosa lacks Peyer's patches and the expression of pIgR (that transports the sIgA into the FRT lumen) depends on the hormonal status of the mice, thus overall IgA titers in vaginal washings can fluctuate. Others have also reported weak and inconsistent sIgA titers in vaginal washings of mice immunized with virus-like particles [37]. Since uterine and vaginal ECs can internalize NPs, it is possible that only internalization by ECs of the upper FRT can lead to priming of local IgA-producing B lymphocytes. In support of this possibility, we observed that vaginally-applied NPs do not always reach the upper FRT. Others have also observed weak or no HIV-1-specific IgA antibodies in humans [38], [39], chimpanzees, and in SIV-infected macaques, while IgG antibodies were detected in sera and external secretions of all species [40], [41], [42], [43]. HIV-positive women with high titers of specific IgG antibodies in sera and cervical secretions exhibited lower genital viral loads compared to women that had only serum antibodies [44], underscoring the importance of local IgG in protection against STIs.

Our finding that NPs administered to the FRT travel to the MLNs where isotype switch can occur, leading to sIgA class switching and subsequent homing of sIgA-producing B cells in the intestinal mucosa, is in line with the common mucosal immune system concept set forth by Bienenstock more than 30 years ago [45]. We also observed that vaginally-administered NPs also reach the large intestine, from where they could be transported to the MLNs. In addition, large intestines contain organized lymphoid tissues similar to Peyer's patches of the small intestine (unpublished observations), thus it is conceivable that the priming of B cells by NPs may occur there. Whether NPs are passively transported to the large intestine and the MLNs via the lymph, or whether there are other means of antigen “shuttling” between the FRT and the intestines remains to be determined.

Another noteworthy observation is that mucosal immunization with NPs induced a Th1/Th2 polarization, while s.c. immunization alone induced mainly Th2 type immune responses, as shown by analysis of serum IgG1 and IgG2c antibody titers. Mucosal priming with NP-Ova appears to be necessary for isotype switching and Th1 polarization, as s.c. immunization with NP-Ova, similarly to s.c. immunization with Ova+CFA, induces high serum IgG1, but not IgG2c (unpublished work). Others have also reported stronger Th1/Th2 polarization in mice that were primed via a mucosal route compared to s.c. primed mice [46]. These finding are very important for developing novel vaccines and prime-boost immunization strategies for induction of protective immunity against mucosal pathogens.

Biodegradable NPs have several advantages that make them attractive for vaccine development. They are internalized efficiently at mucosal surfaces, they are non-infectious and easy to administer, and thus raise no safety concerns. In addition, cocktails of NPs conjugated to a variety of protective antigens can be administered simultaneously for targeting multiple pathogens. NPs used in our work are not biodegradable. However, in more than 10 long-term studies, we have observed no mortality and no adverse effects of NP administration on the health of animals. We are currently investigating the immunogenicity of biodegradable NPs, which in time will replace the use of polystyrene NPs. This work will be important for the development of more effective, needle-free, safe, and affordable mucosal vaccines and therapies. Mucosally-administered vaccines are especially appealing for mass immunization of populations in developing countries with a high prevalence of mucosally-acquired pathogens, including HIV.

Understanding the modes of antigen uptake at mucosal surfaces, antigen transport to the deeper lymphoid tissues, as well as better understanding mucosal memory T and B cell differentiation will aid in optimizing prime-boost immunization strategies for the development of effective mucosal vaccines.

## Supporting Information

S1 Figure **Vaginally-administered 20 nm NPs reach the serosa of the uterus.** (A) An image of the H&E-stained uterine tissue section. (B) A three-color IFM image of the uterine tissue section stained with actin-binding phalloidin-Alexa350 (blue), CD11c antibodies (green), while NPs are shown in red. (C) A high magnification (630x) image of the boxed inset from panel B depicting clumps of NPs within uterine serosa.
(TIF)

S2 Figure **Vaginally-administered NPs reach the mesenteric lymph nodes (MLNs) within 1 h of administration.** (A, B) Confocal images of explanted MLNs 1 h after vaginal NP administration showing NPs (red) within adipose tissue surrounding the MLNs (A) and within the MLN tissue (B). In confocal images, red channel shows NPs, while transmitted light detection was used to visualize the tissue. (C) IFM image of an MLN tissue section harboring NPs (arrows) at 12 h after vaginal NP administration. Tissue section was stained with actin-binding phalloidin-Alexa350 (blue) and NPs are shown in red.
(TIF)

S3 Figure **Dot-blot analysis of serum and fecal extracts of mice vaginally-primed with 20 nm NP-Ova and s.c. boosted with 300 µg Ova with CFA.** After vaginal immunization, mice were fitted with Elizabethan neck collars. 1 week after priming, serum samples of all 6 mice were analyzed (1–6). IgG1columns: Ova (or PBS) were spotted onto nylon membranes, which were then incubated with sera. SIgA column: Nylon membranes with spotted Ova or PBS were blotted with fecal extracts collected from individual mice at day 42. Membranes were then incubated with AP-conjugated goat anti-mouse IgG1 or IgA. Immunoreactive dots were detected by the addition of BCIP. Images were acquired with a digital camera (IgG1) or at 2.5x using a microscope (for IgA).
(TIF)

## References

[1] CDC website**.** Available: http://www.cdc.gov/std/stats/sti-estimates-fact-sheet-feb-2013.pdf. Accessed 2014 Nov 13.

[2] Schiller JT, Castellsague X, Villa LL, Hildesheim A (2008) An update of prophylactic human papillomavirus L1 virus-like particle vaccine clinical trial results. Vaccine 26 Suppl 10: K53–61. 18847557 10.1016/j.vaccine.2008.06.002 PMC2631230

[3] Chachu KA, Strong DW, LoBue AD, Wobus CE, Baric RS, et al (2008) Antibody is critical for the clearance of murine norovirus infection. Journal of virology 82:6610–6617. 18417579 10.1128/JVI.00141-08 PMC2447077

[4] Youngman KR, Franco MA, Kuklin NA, Rott LS, Butcher EC, et al (2002) Correlation of tissue distribution, developmental phenotype, and intestinal homing receptor expression of antigen-specific B cells during the murine anti-rotavirus immune response. Journal of immunology 168:2173–2181. 10.4049/jimmunol.168.5.2173 11859103

[5] Cuff CF, Lavi E, Cebra CK, Cebra JJ, Rubin DH (1990) Passive immunity to fatal reovirus serotype 3-induced meningoencephalitis mediated by both secretory and transplacental factors in neonatal mice. Journal of virology 64:1256–1263. 2154608 10.1128/jvi.64.3.1256-1263.1990 PMC249241

[6] Baba TW, Liska V, Hofmann-Lehmann R, Vlasak J, Xu W, et al (2000) Human neutralizing monoclonal antibodies of the IgG1 subtype protect against mucosal simian-human immunodeficiency virus infection. Nature medicine 6:200–206. 10.1038/72309 10655110

[7] Mascola JR, Stiegler G, VanCott TC, Katinger H, Carpenter CB, et al (2000) Protection of macaques against vaginal transmission of a pathogenic HIV-1/SIV chimeric virus by passive infusion of neutralizing antibodies. Nature medicine 6:207–210. 10.1038/72318 10655111

[8] Veazey RS, Shattock RJ, Pope M, Kirijan JC, Jones J, et al (2003) Prevention of virus transmission to macaque monkeys by a vaginally applied monoclonal antibody to HIV-1 gp120. Nature medicine 9:343–346. 10.1038/nm833 12579198

[9] Wegmann F (2011) Mucosally-targeted HIV-1 vaccines. Human vaccines 7:982–985. 21892008 10.4161/hv.7.9.16505

[10] Lycke N (2012) Recent progress in mucosal vaccine development: potential and limitations. Nature reviews Immunology 12:592–605. 10.1038/nri3251 22828912

[11] Marks E, Helgeby A, Andersson JO, Schon K, Lycke NY (2011) CD4(+) T-cell immunity in the female genital tract is critically dependent on local mucosal immunization. European journal of immunology 41:2642–2653. 21681740 10.1002/eji.201041297

[12] Cuburu N, Graham BS, Buck CB, Kines RC, Pang YY, et al (2012) Intravaginal immunization with HPV vectors induces tissue-resident CD8+ T cell responses. The Journal of clinical investigation 122:4606–4620. 23143305 10.1172/JCI63287 PMC3533540

[13] Owens RB (1974) Glandular epithelial cells from mice: a method for selective cultivation. Journal of the National Cancer Institute 52:1375–1378. 4363715 10.1093/jnci/52.4.1375

[14] Kraehenbuhl JP, Neutra MR (1992) Molecular and cellular basis of immune protection of mucosal surfaces. Physiological reviews 72:853–879. 1438580 10.1152/physrev.1992.72.4.853

[15] Gebert A, Rothkotter HJ, Pabst R (1996) M cells in Peyer's patches of the intestine. International review of cytology 167:91–159. 8768493 10.1016/s0074-7696(08)61346-7

[16] McDole JR, Wheeler LW, McDonald KG, Wang B, Konjufca V, et al (2012) Goblet cells deliver luminal antigen to CD103+ dendritic cells in the small intestine. Nature 483:345–349. 22422267 10.1038/nature10863 PMC3313460

[17] Howe SE, Lickteig DJ, Plunkett KN, Ryerse JS, Konjufca V (2014) The uptake of soluble and particulate antigens by epithelial cells in the mouse small intestine. PloS one 9:e86656. 24475164 10.1371/journal.pone.0086656 PMC3903549

[18] Rescigno M, Urbano M, Valzasina B, Francolini M, Rotta G, et al (2001) Dendritic cells express tight junction proteins and penetrate gut epithelial monolayers to sample bacteria. Nature immunology 2:361–367. 11276208 10.1038/86373

[19] Niess JH, Brand S, Gu X, Landsman L, Jung S, et al (2005) CX3CR1-mediated dendritic cell access to the intestinal lumen and bacterial clearance. Science 307:254–258. 15653504 10.1126/science.1102901

[20] Farache J, Koren I, Milo I, Gurevich I, Kim KW, et al (2013) Luminal bacteria recruit CD103+ dendritic cells into the intestinal epithelium to sample bacterial antigens for presentation. Immunity 38:581–595. 23395676 10.1016/j.immuni.2013.01.009 PMC4115273

[21] Naz RK (2012) Female genital tract immunity: distinct immunological challenges for vaccine development. Journal of reproductive immunology 93:1–8. 22154945 10.1016/j.jri.2011.09.005

[22] Russell MW, Mestecky J (2002) Humoral immune responses to microbial infections in the genital tract. Microbes and infection/Institut Pasteur 4:667–677. 10.1016/s1286-4579(02)01585-x 12048036

[23] Lewis DJ, Fraser CA, Mahmoud AN, Wiggins RC, Woodrow M, et al (2011) Phase I randomised clinical trial of an HIV-1(CN54), clade C, trimeric envelope vaccine candidate delivered vaginally. PloS one 6:e25165. 21984924 10.1371/journal.pone.0025165 PMC3184147

[24] Kozlowski PA, Cu-Uvin S, Neutra MR, Flanigan TP (1997) Comparison of the oral, rectal, and vaginal immunization routes for induction of antibodies in rectal and genital tract secretions of women. Infection and immunity 65:1387–1394. 9119478 10.1128/iai.65.4.1387-1394.1997 PMC175144

[25] Kozlowski PA, Williams SB, Lynch RM, Flanigan TP, Patterson RR, et al (2002) Differential induction of mucosal and systemic antibody responses in women after nasal, rectal, or vaginal immunization: influence of the menstrual cycle. Journal of immunology 169:566–574. 10.4049/jimmunol.169.1.566 12077289

[26] Gillgrass AE, Ashkar AA, Rosenthal KL, Kaushic C (2003) Prolonged exposure to progesterone prevents induction of protective mucosal responses following intravaginal immunization with attenuated herpes simplex virus type 2. Journal of virology 77:9845–9851. 12941893 10.1128/JVI.77.18.9845-9851.2003 PMC224606

[27] Kaushic C, Ashkar AA, Reid LA, Rosenthal KL (2003) Progesterone increases susceptibility and decreases immune responses to genital herpes infection. Journal of virology 77:4558–4565. 12663762 10.1128/JVI.77.8.4558-4565.2003 PMC152159

[28] Cu Y, Booth CJ, Saltzman WM (2011) In vivo distribution of surface-modified PLGA nanoparticles following intravaginal delivery. Journal of controlled release: official journal of the Controlled Release Society 156:258–264. 21763739 10.1016/j.jconrel.2011.06.036 PMC3220785

[29] Ballou B, Andreko SK, Osuna-Highley E, McRaven M, Catalone T, et al (2012) Nanoparticle transport from mouse vagina to adjacent lymph nodes. PloS one 7:e51995. 23284844 10.1371/journal.pone.0051995 PMC3528720

[30] Macpherson AJ, Slack E (2007) The functional interactions of commensal bacteria with intestinal secretory IgA. Current opinion in gastroenterology 23:673–678. 17906446 10.1097/MOG.0b013e3282f0d012

[31] Mestecky J, Moldoveanu Z, Smith PD, Hel Z, Alexander RC (2009) Mucosal immunology of the genital and gastrointestinal tracts and HIV-1 infection. Journal of reproductive immunology 83:196–200. 19853927 10.1016/j.jri.2009.07.005 PMC2802574

[32] Russell MW (2002) Immunization for protection of the reproductive tract: a review. American journal of reproductive immunology 47:265–268. 12148540 10.1034/j.1600-0897.2002.01099.x

[33] Kaul R, Pettengell C, Sheth PM, Sunderji S, Biringer A, et al (2008) The genital tract immune milieu: an important determinant of HIV susceptibility and secondary transmission. Journal of reproductive immunology 77:32–40. 17395270 10.1016/j.jri.2007.02.002

[34] Schleiss MR, Bourne N, Jensen NJ, Bravo F, Bernstein DI (2000) Immunogenicity evaluation of DNA vaccines that target guinea pig cytomegalovirus proteins glycoprotein B and UL83. Viral immunology 13:155–167. 10892996 10.1089/vim.2000.13.155

[35] Mestecky J (2007) Humoral immune responses to the human immunodeficiency virus type-1 (HIV-1) in the genital tract compared to other mucosal sites. Journal of reproductive immunology 73:86–97. 17354294 10.1016/j.jri.2007.01.006

[36] Mestecky J, Moldoveanu Z, Russell MW (2005) Immunologic uniqueness of the genital tract: challenge for vaccine development. American journal of reproductive immunology 53:208–214. 15833098 10.1111/j.1600-0897.2005.00267.x

[37] Hunter Z, Tumban E, Dziduszko A, Chackerian B (2011) Aerosol delivery of virus-like particles to the genital tract induces local and systemic antibody responses. Vaccine 29:4584–4592. 21549786 10.1016/j.vaccine.2011.04.051 PMC3114090

[38] Dorrell L, Hessell AJ, Wang M, Whittle H, Sabally S, et al (2000) Absence of specific mucosal antibody responses in HIV-exposed uninfected sex workers from the Gambia. AIDS 14:1117–1122. 10894275 10.1097/00002030-200006160-00008

[39] Skurnick JH, Palumbo P, DeVico A, Shacklett BL, Valentine FT, et al (2002) Correlates of nontransmission in US women at high risk of human immunodeficiency virus type 1 infection through sexual exposure. The Journal of infectious diseases 185:428–438. 11865394 10.1086/338830 PMC2743095

[40] Wright PF, Kozlowski PA, Rybczyk GK, Goepfert P, Staats HF, et al (2002) Detection of mucosal antibodies in HIV type 1-infected individuals. AIDS research and human retroviruses 18:1291–1300. 12487817 10.1089/088922202320886334

[41] Mestecky J, Jackson S, Moldoveanu Z, Nesbit LR, Kulhavy R, et al (2004) Paucity of antigen-specific IgA responses in sera and external secretions of HIV-type 1-infected individuals. AIDS research and human retroviruses 20:972–988. 15585085 10.1089/aid.2004.20.972

[42] Raux M, Finkielsztejn L, Salmon-Ceron D, Bouchez H, Excler JL, et al (1999) Comparison of the distribution of IgG and IgA antibodies in serum and various mucosal fluids of HIV type 1-infected subjects. AIDS research and human retroviruses 15:1365–1376. 10515152 10.1089/088922299310070

[43] Schafer F, Kewenig S, Stolte N, Stahl-Hennig C, Stallmach A, et al (2002) Lack of simian immunodeficiency virus (SIV) specific IgA response in the intestine of SIV infected rhesus macaques. Gut 50:608–614. 11950804 10.1136/gut.50.5.608 PMC1773198

[44] Nag P, Kim J, Sapiega V, Landay AL, Bremer JW, et al (2004) Women with cervicovaginal antibody-dependent cell-mediated cytotoxicity have lower genital HIV-1 RNA loads. The Journal of infectious diseases 190:1970–1978. 15529262 10.1086/425582 PMC3119045

[45] Bienenstock J, McDermott M, Befus D, O'Neill M (1978) A common mucosal immunologic system involving the bronchus, breast and bowel. Advances in experimental medicine and biology 107:53–59. 742502 10.1007/978-1-4684-3369-2_7

[46] Fiorino F, Pettini E, Pozzi G, Medaglini D, Ciabattini A (2013) Prime-boost strategies in mucosal immunization affect local IgA production and the type of th response. Frontiers in immunology 4:128. 23755051 10.3389/fimmu.2013.00128 PMC3665932

