# Supplementary figures and images for: Protein-Coated Nanoparticles Are Internalized by the Epithelial Cells of the Female Reproductive Tract and Induce Systemic and Mucosal Immune Responses
Source: PLoS One. 2014 Dec 9;9(12):e114601. doi: 10.1371/journal.pone.0114601 (PMC4260873; doi:10.1371/journal.pone.0114601)

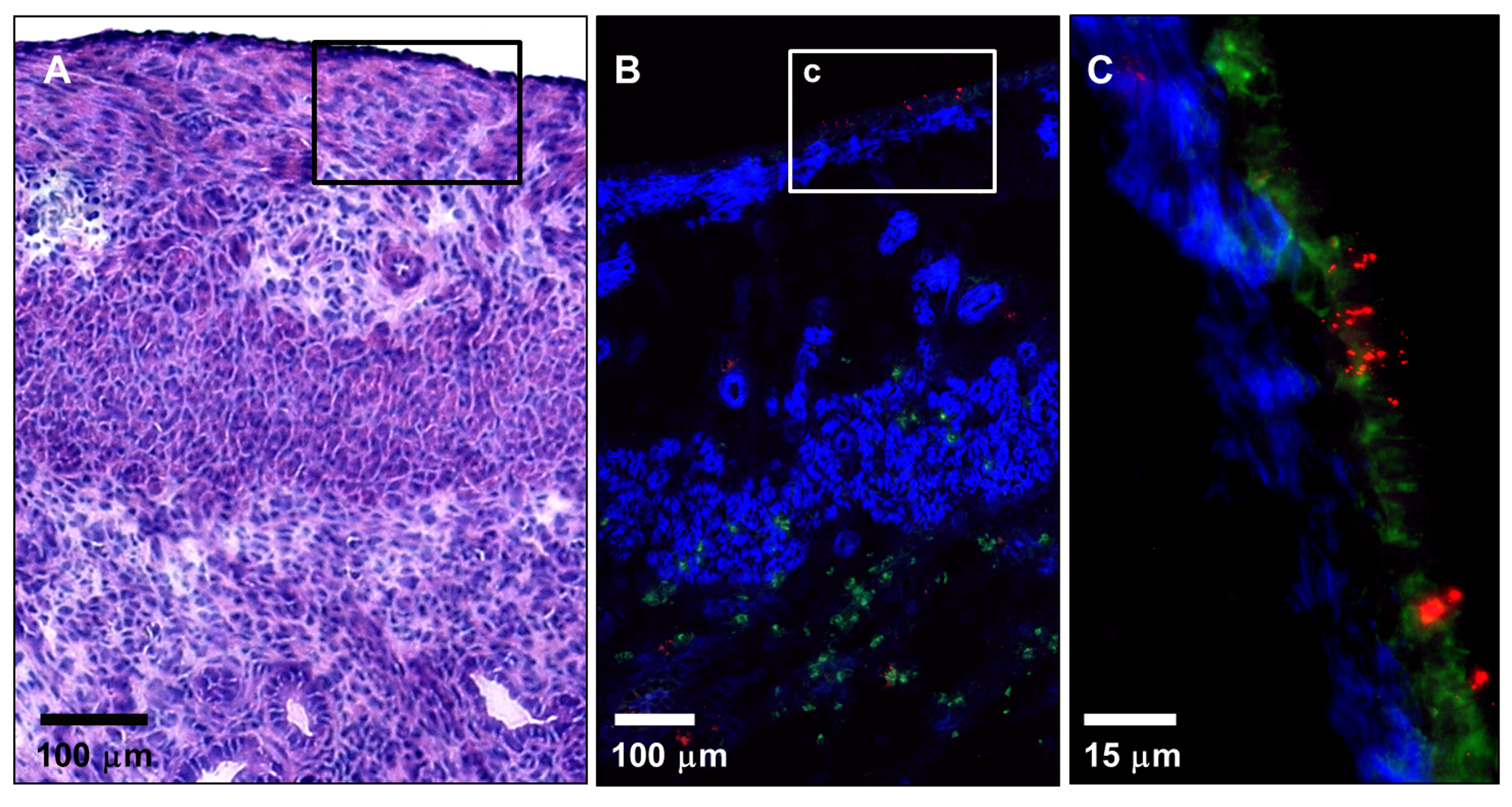

Supplement: S1 Figure — Vaginally-administered 20 nm NPs reach the serosa of the uterus. (A) An image of the H&E-stained uterine tissue section. (B) A three-color IFM image of the uterine tissue section stained with actin-binding phalloidin-Alexa350 (blue), CD11c antibodies (green), while NPs are shown in red. (C) A high magnification (630x) image of the boxed inset from panel B depicting clumps of NPs within uterine serosa. (TIF) [file pone.0114601.s001.tif]

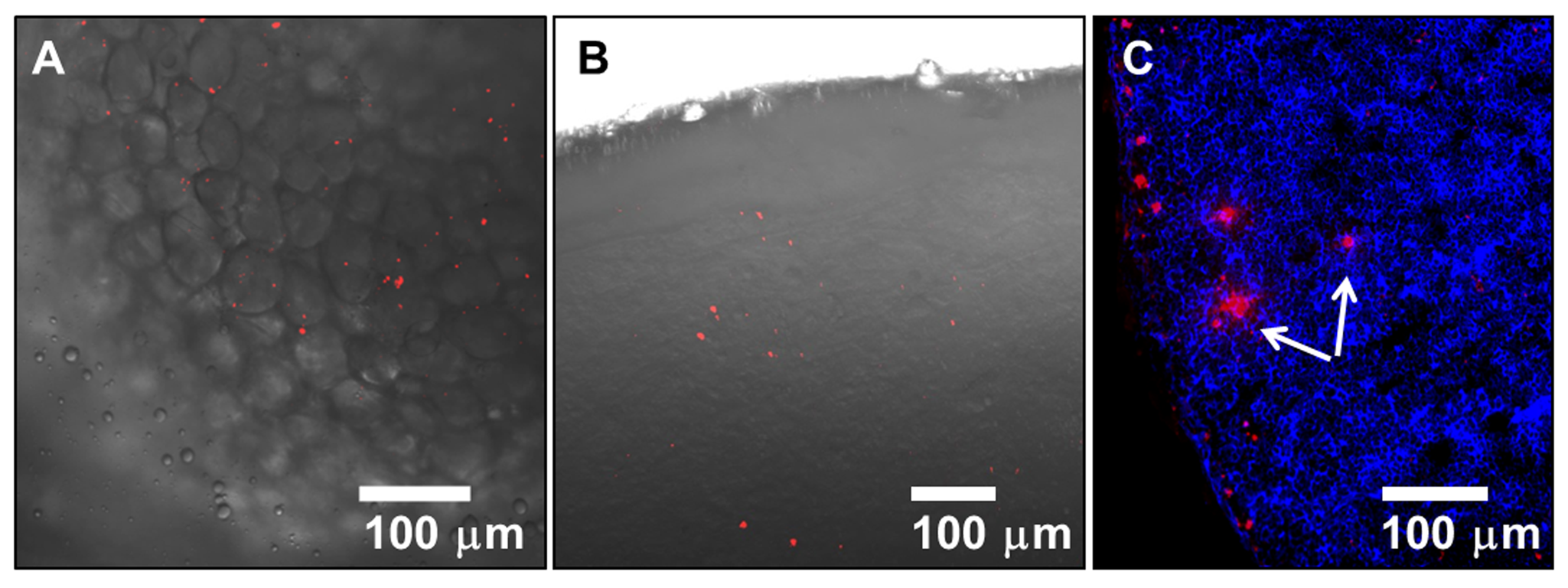

Supplement: S2 Figure — Vaginally-administered NPs reach the mesenteric lymph nodes (MLNs) within 1 h of administration. (A, B) Confocal images of explanted MLNs 1 h after vaginal NP administration showing NPs (red) within adipose tissue surrounding the MLNs (A) and within the MLN tissue (B). In confocal images, red channel shows NPs, while transmitted light detection was used to visualize the tissue. (C) IFM image of an MLN tissue section harboring NPs (arrows) at 12 h after vaginal NP administration. Tissue section was stained with actin-binding phalloidin-Alexa350 (blue) and NPs are shown in red. (TIF) [file pone.0114601.s002.tif]

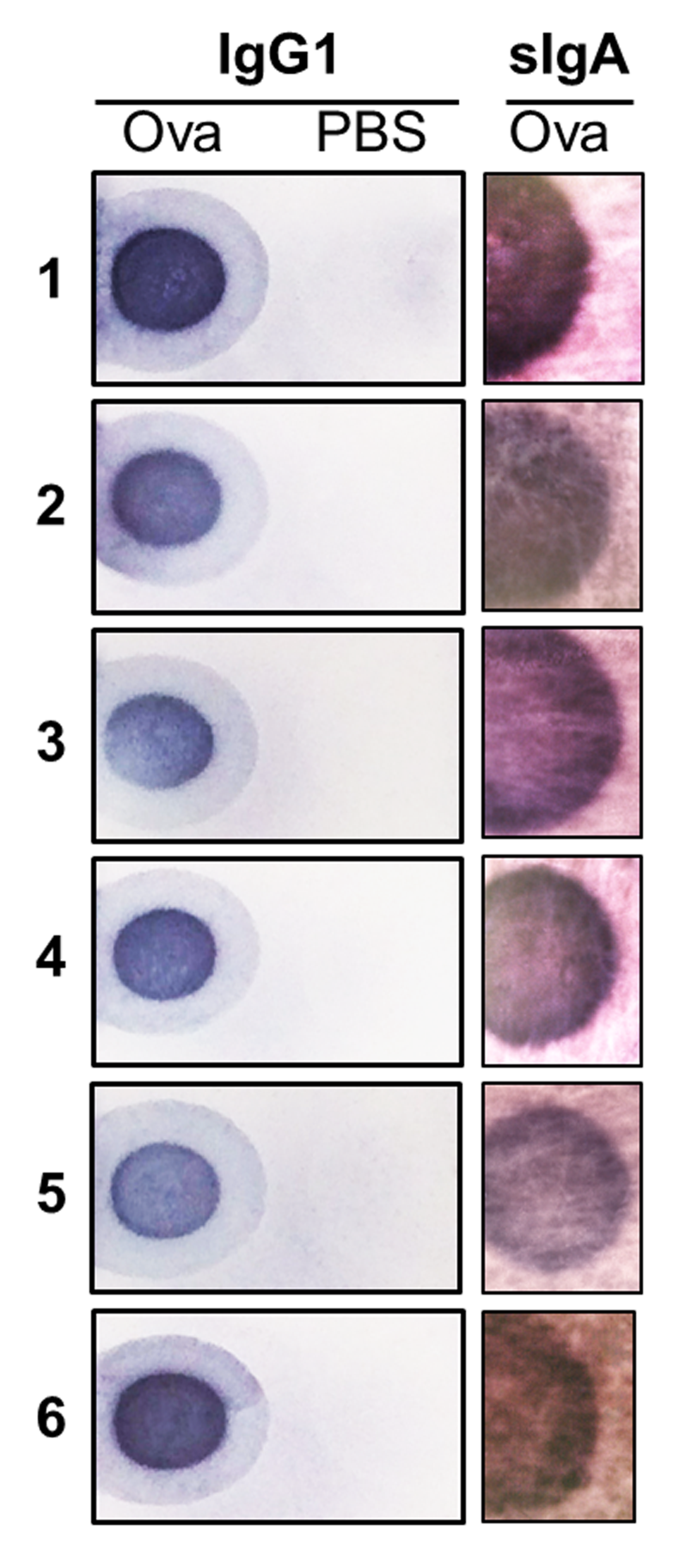

Supplement: S3 Figure — Dot-blot analysis of serum and fecal extracts of mice vaginally-primed with 20 nm NP-Ova and s.c. boosted with 300 µg Ova with CFA. After vaginal immunization, mice were fitted with Elizabethan neck collars. 1 week after priming, serum samples of all 6 mice were analyzed (1–6). IgG1columns: Ova (or PBS) were spotted onto nylon membranes, which were then incubated with sera. SIgA column: Nylon membranes with spotted Ova or PBS were blotted with fecal extracts collected from individual mice at day 42. Membranes were then incubated with AP-conjugated goat anti-mouse IgG1 or IgA. Immunoreactive dots were detected by the addition of BCIP. Images were acquired with a digital camera (IgG1) or at 2.5x using a microscope (for IgA). (TIF) [file pone.0114601.s003.tif]
